# Complex Relationship of Left Ventricular Rotational Mechanics and Deformation Represented by Strain Parameters in Healthy Adults—Detailed Analysis from the Three-Dimensional Speckle-Tracking Echocardiographic MAGYAR-Healthy Study

**DOI:** 10.3390/jcm12237389

**Published:** 2023-11-29

**Authors:** Attila Nemes, Árpád Kormányos, Nóra Ambrus, Csaba Lengyel

**Affiliations:** Department of Medicine, Albert Szent-Györgyi Medical School, University of Szeged, H-6725 Szeged, Hungary; kormanyos.arpad@med.u-szeged.hu (Á.K.); ambrus.nora@med.u-szeged.hu (N.A.); lengyel.csaba@med.u-szeged.hu (C.L.)

**Keywords:** left ventricular, rotation, strain, three-dimensional, speckle-tracking, echocardiography

## Abstract

Introduction: Left ventricular (LV) strains are measures of deformation that reflect LV function quantifying the rate of LV contraction, providing information in three directions in space: radial (RS), longitudinal (LS) and circumferential directions (CS). The LV moves around its longitudinal axis in a special movement called LV rotational mechanics. The present study aimed to assess associations between three-dimensional speckle-tracking echocardiography (3DSTE)-derived LV rotational mechanics and LV strains in healthy adult subjects. Methods: The present study consisted of 174 healthy adults (mean age: 32.8 ± 12.2 years, 79 males). Complete two-dimensional Doppler echocardiography and 3DSTE were performed in all subjects. Results: While LV-gRS and LV-gLS did not show associations with increased basal LV rotation, the lowest LV-gCS was seen in the presence of the highest LV basal rotation. An increase in basal LV rotation and consequential LV twist were not associated with apical LV rotation. While LV-gLS was not associated with the increase in apical LV rotation, LV-gRS and LV-gCS showed a trend towards increasing values. An increase in LV-gRS was associated with an increasing trend towards apical LV rotation, LV twist and LV-gCS and the preservation of basal LV rotation. LV-gLS also increased but only up to a certain value. An increase in LV-gCS was associated with a tendency towards a decrease in basal LV rotation and a tendency towards an increase in LV-gRS and LV-gLS. The highest LV-gCS was associated with the highest apical LV rotation and LV twist. The highest apical LV rotation, LV twist and LV-gCS were seen in the presence of the highest LV-gLS, while basal LV rotation and LV-gRS were not associated with increasing LV-gLS. Conclusions: Basal LV rotation has been shown to have an inverse relationship with LV-gCS, but without being related to LV-gRS and LV-gLS, while apical LV rotation is associated with LV strains in all directions, but to a different extent, suggesting a complex relationship between LV rotational mechanics and LV strains in healthy adults.

## 1. Introduction

During the heart cycle, the left ventricular (LV) wall not only contracts but also twists at the same time, maximizing its functional effectivity, intracavitary pressure and stroke volume and minimizing myocardial oxygen demand [1,2,3,4,5,6,7,8,9]. LV strains are measures of deformation that reflect LV function quantifying the rate of LV contraction, providing information in three directions in space: radial (RS), longitudinal (LS) and circumferential directions (CS) [6,7,8,9]. However, there is a special component of the movement of the LV around its longitudinal axis called LV rotational mechanics. The movement is similar to wringing out a towel and is called LV twist, which represents the absolute difference in the magnitude of apical and basal LV rotations [1,2,3,4,5,6]. Understanding the complexity of the above-mentioned LV movements with respect to the heart cycle has both physiological and clinical significance. Abnormalities of LV deformation represented by strains and LV rotational mechanics in certain pathologies have been widely examined [5,6,10]. Although the widely used two-dimensional (2D) speckle-tracking echocardiography (STE) can be used for quantitative characterization of LV rotational mechanics and strains [8,11], it does not reliably measure LV twist [12]. However, three-dimensional (3D) STE seems to have potential for simultaneous assessment of LV rotational parameters and strains [13,14,15,16,17]. 3DSTE offers validated, sensitive and reproducible measurements of these parameters [18,19,20,21,22,23]. The present study aimed to assess associations between different grades of 3DSTE-derived LV rotational mechanics and LV strains in healthy adult individuals.

## 2. Subjects and Methods

### 2.1. Subject Population

The present study comprised 174 healthy adults (average age: 32.8 ± 12.2 years, 79 males). Volunteers without any known disorders or pathological states were involved between 2011 and 2015. They underwent physical examination, laboratory assessments, standard 12-lead electrocardiography (ECG) and 2D Doppler echocardiography, which was followed by 3DSTE. All tests ended with negative results, which were within the normal reference range. None of the participants was an athlete or obese or took regular medication. The analysis carried out in this study is part of the ‘Motion analysis of the heart and great vessels by three-dimensional speckle-tracking echocardiography in healthy subjects’ (MAGYAR-Healthy) study, which has been organized to examine physiological relationships under healthy circumstances (‘Magyar’ means ‘Hungarian’ in the Hungarian language). This investigation was conducted in accordance with the Declaration of Helsinki (as revised in 2013). The Institutional and Regional Human Biomedical Research Committee at the University of Szeged, Hungary (registration number: 71/2011 and updated versions) approved the study. Informed consent was given by all volunteers.

### 2.2. Two-Dimensional Doppler Echocardiography

During the 2D echocardiographic studies, we followed the accepted professional guidelines [24]. According to our practice, a complete 2D echocardiographic examination was performed with Doppler analysis in all subjects first. During the tests, the patient was asked to lie on his left side, and then the heart was examined in the typical parasternal and apical views. Chamber quantifications were carried out in accordance with the guidelines, including the determination of the LV ejection fraction by Simpson’s method. Significant valvular stenoses and insufficiencies were excluded by Doppler echocardiography. Pulsed Doppler helped the measurement of transmitral flow velocities in diastole and their ratio (E/A). For all analysis, the Toshiba Artida^TM^ echocardiographic machine (Toshiba Medical Systems, Tokyo, Japan) was used in all healthy subjects, attached to a PST-30BT (1–5 MHz) phased-array transducer.

### 2.3. Three-Dimensional Speckle-Tracking Echocardiography

Immediately after the 2D echocardiographic examination, 3DSTE was performed. All 3DSTE analyses followed our standard protocol. Firstly, in healthy subjects in the left lateral decubitus position, 3D echocardiographic data acquisitions were performed from an apical window following image optimizations (gain, magnitude, etc.) with the same Toshiba Artida^TM^ echocardiographic tool (Toshiba Medical Systems, Tokyo, Japan), but using a 3D-capable PST-25SX matrix-array transducer. ECG gating was used for all subjects who were in sinus rhythm, during six cardiac cycles; one wedge-shaped subvolume per cardiac cycle was acquired to achieve the best image quality. The software stitched them together, creating a pyramid-shaped full-volume 3D echocardiographic dataset called an ‘echocloud’ [13,14,15,16,17].

Secondly, at a later date, 3D echocardiographic datasets were analysed offline with 3D Wall Motion Tracking software (Ultra Extend, Toshiba Medical Systems, Tokyo, Japan, version 2.7, vendor-provided). The aim of the analysis was to create a 3D model/cast of the LV for same-time determination of LV strains and rotational parameters. During the analysis, the software automatically created a five-division figure with apical longitudinal four-chamber (AP4CH) and two-chamber (AP2CH) views and basal, midventricular and apical cross-sectional views. For the creation of the LV 3D cast, septal and lateral mitral annular (MA)-LV edges and the apical endocardial surface of the LV were defined, after which a sequential analysis was performed [13,14,15,16,17].

**Figure 1:**
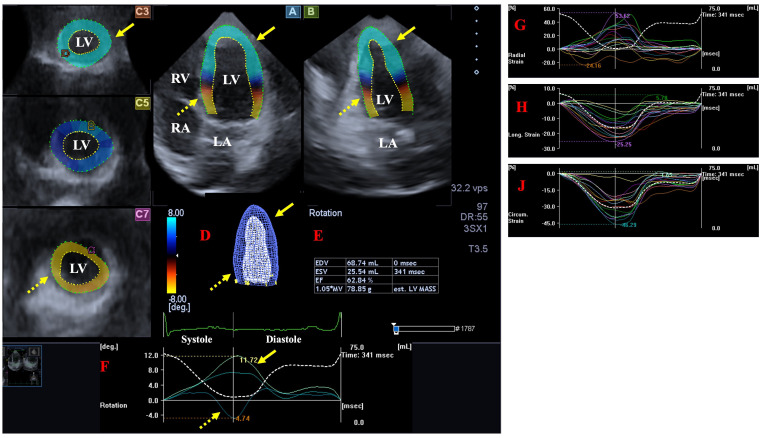
Three-dimensional (3D) speckle-tracking echocardiographic examination of the left ventricle (LV) in a healthy subject: apical longitudinal four-chamber (**A**) and two-chamber views (**B**) and apical (**C3**), midventricular (**C5**) and basal (**C7**) short-axis views are demonstrated together with 3D cast of the LV (**D**), volumetric LV parameters and LV ejection fraction (**E**) and time-LV volume changes (dashed white line). Time—apical (yellow arrow) and basal (dashed yellow arrow) LV rotations (**F**) and global (white line) and segmental (coloured lines) time—radial (**G**), longitudinal (**H**) and circumferential (**J**) LV strain curves are also shown. Abbreviations. LV = left ventricle, LA = left atrium, RA = right atrium, RV = right ventricle.

Thirdly, using this 3D LV model, several functional LV properties were calculated (Figure 1):

- The following LV rotational parameters were provided: apical and basal LV rotations, LV twist and time-to-peak LV twist [25].
- The following global LV strains, featuring the whole LV were measured [26]:
  ○ LV radial strain (LV-gRS)—for characterizing thinning/thickening of the myocardial tissue.
  ○ LV longitudinal strain (LV-gLS)—for characterizing lengthening/shortening of the myocardial tissue.
  ○ LV circumferential strain (LV-gCS)—for characterizing widening/narrowing of the myocardial tissue.

### 2.4. Statistical Analysis

Mean ± standard deviation and *n* (%) formats were used for data presentations, as appropriate. Significance was determined when *p* was less than 0.05. Depending on whether the data were continuous or categorical, Student’s *t*-test, Bonferroni method following one-way analysis of variance (ANOVA) and Fischer’s exact test were used, where appropriate. Statistical analyses were performed with SPSS version 22 (SPSS Inc., Michigan, IL, USA) software.

## 3. Results

### 3.1. Clinical Data 

The most important clinical parameters were within the normal ranges, including systolic (122.3 ± 5.1 mm Hg) and diastolic (75.7 ± 4.6 mm Hg) blood pressures and heart rate (71.9 ± 2.8 1/s). The mean height, weight, calculated body surface area and body mass index were 170.5 ± 7.6 cm, 72.1 ± 10.9 kg, 1.87 ± 0.13 m^2^ and 24.5 ± 2.3 kg/m^2^, respectively.

### 3.2. Two-Dimensional Doppler Echocardiography

Routine 2D echocardiographic parameters proved to be normal: left atrial diameter assessed in parasternal long-axis view (36.5 ± 3.9 mm), LV end-diastolic and end-systolic diameters (48.1 ± 3.7 mm and 32.0 ± 3.4 mm, respectively), LV end-diastolic and end-systolic volumes (106.9 ± 22.8 mL and 36.5 ± 9.2 mL, respectively), interventricular septum (9.0 ± 1.5 mm) and LV posterior wall (9.1 ± 1.6 mm) and LV ejection fraction (65.9 ± 4.9%). Mean transmitral flow velocities measured in diastole were 79.3 ± 17.5 cm/s and 63.4 ± 19.3 cm/s, respectively. None of the participants had larger than grade 1 valvular regurgitation or showed valvular stenosis on any valves.

### 3.3. Three-Dimensional Speckle-Tracking Echocardiography

Using the same 3D cast of the LV, the following LV parameters were calculated: LV-EDV, LV-ESV, LV-EF, LV mass, basal and apical LV rotations. LV-gRS, LV-gCS and LV-gLS proved to be 86.2 ± 23.1 mL, 36.3 ± 10.5 mL, 58.2 ± 5.6%, 158.2 ± 31.8 g, −4.10 ± 2.07 degree, 9.31 ± 3.79 degree, 25.4 ± 9.3%, −27.6 ± 5.0% and −16.1 ± 2.5%, respectively. 3DSTE-derived maximum, pre-atrial contraction and minimum left atrial volumes proved to be 40.5 ± 12.5 mL, 27. 5 ± 10.9 mL and 19.1 ± 7.8 mL, respectively.

### 3.4. Classification of Subjects

Subgroups were created based on mean ± SD of basal and apical LV rotations and LV-gRS, LV-gCS and LV-gLS. Subjects were classified into these subgroups according to the lower (2.03 degree, 5.60 degree, 16.1%, −22.6% and −13.6%, respectively) and upper (6.17 degree, 13.02 degree, 34.7%, −22.6% and −18.6%, respectively) values of the above-mentioned variables.

### 3.5. Increasing LV Apical and Basal Rotations and LV Strains

While LV-gRS and LV-gLS did not show any associations with the increase in basal LV rotation, the lowest LV-gCS was seen in the presence of the highest LV basal rotation. An increase in basal LV rotation and consequential LV twist were not associated with apical LV rotation. While LV-gLS was not associated with an increase in apical LV rotation, LV-gRS and LV-gCS showed a tendency towards an increase (Table 1).

### 3.6. Increasing LV Strains and LV Apical and Basal Rotations

An increase in LV-gRS was associated with an increasing trend towards apical LV rotation, LV twist and LV-gCS and the preservation of basal LV rotation. LV-gLS increased, but only up to a certain value. An increase in LV-gCS was associated with a tendency towards decrease in basal LV rotation and a tendency towards an increase in LV-gRS and LV-gLS. The highest LV-gCS was associated with the highest apical LV rotation and LV twist. The highest apical LV rotation, LV twist and LV-gCS were seen in the presence of the highest LV-gLS, while basal LV rotation and LV-gRS were not associated with an increase in LV-gLS (Table 2).

### 3.7. Feasibility of 3DSTE-Derived LV Quantifications

Adequate evaluations could be carried out in 174 out of 298 subjects (58% success ratio).

## 4. Discussion

The structure of the heart muscle is very specialized. Numerous experimental and clinical studies have clarified its structure [3,8,27,28,29,30]. In brief, the LV muscle fibers in the subepicardium run in a left-handed direction, the fibers of the middle layer run circumferentially, while the fibers of the subendocardium run in a right-handed direction. According to the above facts, the contraction of the subendocardium and the subepicardium would result in rotation in opposite directions; however, the rotational radius of the subepicardium is larger than that of the subendocardium, hence exerting greater torque, as a result of which the rotation of the subepicardium is significantly expressed. At the same time, owing to the spiral architecture of the muscle fibers, a wringing-like motion of the LV could be detected in systole during ejection. Because myocardial fibers on the subepicardial side run in left-handed direction, there is a clockwise rotation at the base of the LV, while counterclockwise rotation occurs at the apex of the LV. That is why hyperrotation is observed in subendocardial dysfunction, while hyporotation is observed in the case of injury to the subepicardium. This sort of movement is called LV twist [1,2,3,4,5,6]. This form of LV function allows LV emptying to be maximized, optimized and fine-tuned [1,2,3,4,5,6]. LV twist is responsible for the fact that a 15–20% shortening of myocardial fibers eventually manifests itself in a 60–70% LV ejection fraction. During diastole, each muscle must return to its starting point, a movement that is called LV untwisting [3].

However, LV not only twists during the heart cycle but also performs a contracting–relaxing motion. Moreover, in real life, LV rotational mechanics cannot be separated from this complex form of LV movement, which occurs in coordination simultaneously. While the LV muscle contracts and thickens radially, there is a shortening in the longitudinal and circumferential (in other words narrowing) dimensions in systole. The myocardial fiber orientation and LV contraction pattern allow the determination of three simple unidimensional-unidirectional strains: longitudinal (LV-LS), circumferential (LV-CS) and radial (LV-RS) strains. Note that modern imaging techniques such as 3DSTE can also compute complex strains calculated as a combination of simple strains, such as area strain (LV-AS, combination of LV-LS and LV-CS) and 3D strain (LV−3DS, combination of LV-LS, LV-CS and LV-RS). The above-mentioned LV radial thickening in systole is represented by LV-RS (positive strain), while longitudinal and circumferential shortening and narrowing are represented by LV-LS and LV-CS, respectively (negative strains) [7,8,13].

In recent decades, cardiovascular imaging has undergone tremendous technical advancement. Methods such as cardiac computer tomography and magnetic resonance imaging have become part of the daily clinical routine, making it possible to diagnose diseases that could not be examined fully or at all until then. But ultrasound technology has also undergone outstanding development. In just over 25 years, completely new cardiovascular ultrasound methods have appeared and spread (e.g., 3D echocardiography and STE), and certain parameters (e.g., global LV-LS) have been included in professional guidelines. Developments have resulted in the ability to analyse the chambers and valves of the heart non-invasively, in a relatively short time, in an easy-to-learn way. Although the emergence of these methods requires a new approach and takes some time to learn, when used in conjunction with previously widespread techniques, they have significantly complemented diagnostic possibilities. Three-dimensional echocardiography has made volumetric measurements more accurate by taking into account the exact shape of the heart cavities using spatial models, while STE allows the contractility of the walls of a heart cavity to be objectified using strain parameters. From a theoretical point of view, a 2D examination of a 3D organ should lead to the loss of certain information. This is where 3DSTE can help, combining the advantages of 3D echocardiography and STE. Although current recommendations do not consider 2DSTE suitable for quantitative characterization of LV rotational mechanics [12], 3DSTE is [13,14,15,16,17]. In short, 3DSTE combines the advantages of both methods, as precise measurement of LV volumetric, strain and rotational parameters can be performed simultaneously during the same test using virtual spatial 3D LV models created in a relatively short time [13,14,15,16,17].

All these abilities give us an examination method that is suitable for performing clinical physiological examinations without harming the interests of our patients; moreover, they can receive detailed information about their cardiac statuses in a non-invasive way. We have the opportunity to compare physiological parameters under healthy conditions. Although the aim of the present study was only to compare the deformation and rotational functional parameters of LV, within the framework of the MAGYAR-Healthy Study, the examination of the relationships of other cavities and valves of the heart and their significance was partly confirmed [25,26]. Based on data in the literature, it can be said that 3DSTE is considered to be validated for the measurement of LV strains [19,20] and rotational parameters [21,22,23] as well.

In recent studies, the dependence of LV rotations on LV volumes has already been demonstrated, together with a complex pattern of LV strains in the presence of higher/lower LV volumes [25,26]. The relationship between volume changes and the contractility pattern was demonstrated to be regulated by the Frank–Starling mechanism [31]. The results of the present study add to this knowledge by showing the complexity of LV rotational mechanics and deformation represented by LV strains in healthy individuals. Basal LV rotation has been shown to have an inverse relationship with LV-gCS, but without being related to LV-gRS and LV-gLS. Apical LV rotation is associated with LV strains in all directions, but to a different extent. These results demonstrate a complex LV contractility and rotational pattern even under healthy circumstances. With better understanding, the complexity of the pattern of LV deformation and rotational mechanics may help to find (combined) forms of movement that may be characteristic of certain diseases. Three-dimensional STE-derived analysis has already been carried out, in which certain associations could be confirmed in terms of volumetric and functional abnormalities linked to the LA [32]. Another well-known example is the use of LV apical sparing in the diagnosis of cardiac amyloidosis [33]. However, further studies are warranted in larger populations including populations with certain pathologies.

## 5. Limitations

The following important limitations have to be mentioned regarding this study:

- Although healthy individuals were included in the study and there were no known factors that could have influenced the findings, it cannot be ruled out 100% that they had some kind of subclinical latent disease. Further laboratory and imaging tests could have ruled them out.
- An important technical problem that still exists today is 3DSTE image quality, which is worse than that of 2D echocardiography due to technical reasons (lower spatial and temporal resolution) [13,14,15,16,17]. During 3DSTE analysis, ECG gating and data acquisition within four–six heart cycles are required to achieve optimal image quality. In addition, to create the 3D full volume, subvolumes are first recorded, which are later stitched together by the software. However, this makes an opportunity to create artifacts. In addition, respiratory movement and arrhythmias can make data acquisition and therefore imaging difficult.
- In the present study, only LV data were analyzed. However, 3DSTE-derived (left and right) atrial analysis could have been performed at the same time, as demonstrated in recent studies [34].
- The investigators did not validate 3DSTE-derived LV strains and rotational parameters due to the fact that this sort of measurement has already been validated [18,19,20,21,22,23].
- Similarly, the determination of 3DSTE-derived normal reference values of LV rotational parameters and strains was not aimed in this paper because they are already defined [35,36].
- Although there may be debate about which chamber the ventricular septum is part of, it was clearly considered part of the LV in the present investigation.
- Three-dimensional STE allows the determination of complex/multidimensional LV strains as well, such as 3D and area strains, which are formed from unidimensional LV strains. However, due to their complex nature, these parameters were not examined.
- LV dimensions and volumes were not involved in the analyses. However, relationships between LV rotational mechanics and strains and LV volumes have already been examined in the frame of the MAGYAR-Healthy Study [25,26].
- The relationship between global LV strains and rotational parameters determined only by 3DSTE was studied. The presentation of other morphological abnormalities of LV, the measurement of the diameters or areas of LV in a particular selected plane or the analysis of segmental or regional strain and rotational parameters of the LV were not the aim of this study.
- This study is a retrospective study, which limits the value of the conclusions that can be drawn.

## 6. Conclusions

Basal LV rotation has been shown to have an inverse relationship with LV-gCS, but without being related to LV-gRS and LV-gLS, while apical LV rotation is associated with LV strains in all directions, but to a different extent, suggesting a complex relationship between LV rotational mechanics and LV strains in healthy adults.

## Figures and Tables

**Table 1:** Left ventricular global strains in different left ventricular rotation groups.

	Basal LV Rotation < 2.03° (*n* = 23)	2.03° ≤ Basal LV Rotation ≤ 6.17° (*n* = 123)	6.17° < Basal LV Rotation (*n* = 28)	Apical LV Rotation < 5.60° (*n* = 25)	5.60° ≤ Apical LV Rotation ≤ 13.02° (*n* = 121)	13.02° < Apical LV Rotation (*n* = 28)
basal LV rotation (°)	−1.33 ± 0.62	−3.75 ± 1.08 *	−7.78 ± 1.02 *^†^	−4.25 ± 2.54	−4.14 ± 1.96	−3.80 ± 2.02
apical LV rotation (°)	9.76 ± 4.31	9.39 ± 3.68	8.52 ± 3.68	3.63 ± 1.51	9.03 ± 1.90 ^§^	15.56 ± 1.86 ^§‡^
LV twist (°)	11.08 ± 4.44	13.14 ± 3.60 *	16.3 ± 3.97 *^†^	7.88 ± 3.01	13.17 ± 2.43 ^§^	19.36 ± 2.5 ^§‡^
time-to-peak LV twist (ms)	297.4 ± 103.8	361.3 ± 137.5 *	342.4 ± 80.8	338.5 ± 125.7	353.3 ± 132.4	345.3 ± 104.6
LV-gRS (%)	24.2 ± 7.3	25.7 ± 9.5	23.9 ± 7.9	23.9 ± 7.6	25.0 ± 8.6	28.6 ± 12.2 ^‡^
LV-gCS (%)	−28.2 ± 3.1	−28.2 ± 4.8	−24.4 ± 5.6 *^†^	−25.2 ± 3.8	−27.5 ± 4.4 ^§^	−30.6 ± 6.5 ^§‡^
LV-gLS (%)	−15.9 ± 2.2	−16.1 ± 2.5	−16.0 ± 2.5	−16.1 ± 1.4	−15.9 ± 2.6	−16.8 ± 2.5

Abbreviations: LV-gRS = LV global radial strain, LV-gCS = LV global circumferential strain, LV-gLS = LV global longitudinal strain, LV-gAS = LV global area strain, LV-g3DS = LV global three-dimensional strain, LV = left ventricular. * *p* < 0.05 vs. basal LVrot < 2.03 degree; ^†^ *p* < 0.05 vs. 2.03 degree ≤ basal LVrot ≤ 6.17 degree; ^§^ *p* < 0.05 vs. apical LVrot < 5.60 degree; ^‡^ *p* < 0.05 vs. 5.60 degree ≤ apical LVrot ≤ 13.02 degree.

**Table 2:** Left ventricular rotational parameters and global strains in different global left ventricular radial, circumferential and longitudinal strain groups.

	LV-gRS < 16.1% (*n* = 26)	16.1% ≤ LV-gRS ≤ 34.70% (*n* = 123)	34.70% < LV-gRS (*n* = 25)	LV-gCS < −22.6% (*n* = 18)	−22.6% ≤ LV-gCS ≤ −32.6% (*n* = 127)	−32.6% < LV-gCS (*n* = 29)	LV-gLS < −13.6% (*n* = 26)	−13.6% ≤ LV-gLS ≤ −18.6% (*n* = 122)	−18.6% < LV-gLS (*n* = 26)
basal LV rotation (°)	−4.13 ± 1.88	−4.12 ± 2.15	4.00 ± 1.83	−5.93 ± 1.94	−3.98 ± 2.05 ^§^	−3.49 ± 1.59 ^§‡^	−3.95 ± 1.82	−4.10 ± 2.15	−4.24 ± 1.92
apical LV rotation (°)	8.12 ± 2.90	9.34 ± 3.93	10.38 ± 3.53 *	8.77 ± 4.17	8.76 ± 3.41	12.05 ± 3.91 ^§‡^	9.43 ± 2.54	8.88 ± 3.91	11.17 ± 3.68 ^#&^
LV twist (°)	12.24 ± 3.58	13.46 ± 4.24	14.37 ± 3.32 *	14.70 ± 4.90	12.74 ± 3.78 ^§^	15.53 ± 3.80^‡^	13.38 ± 3.10	12.98 ± 4.26	15.41 ± 3.36 ^#^
time-to-peak LV twist (ms)	340.0 ± 171.1	348.5 ± 118.2	367.4 ± 115.7	318.1 ± 46.7	357.7 ± 135.0	334.6 ± 121.5 ^‡^	325.8 ± 145.1	361.4 ± 131.4	320.8 ± 69.1
LV-gRS (%)	12.8 ± 3.1	24.7 ± 4.6 *	42.1 ± 6.5 *^†^	20.6 ± 8.3	24.4 ± 8.1 ^§^	33.0 ± 10.5 ^§‡^	26.6 ± 11.8	24.6 ± 8.4	27.9 ± 9.9
LV-gCS (%)	−25.2 ± 4.3	−27.5 ± 4.6 *	−30.7 ± 5.9 *^†^	−19.9 ± 2.3	−26.9 ± 2.6 ^§^	−35.9 ± 2.7 ^§‡^	−27.0 ± 4.4	−27.2 ± 4.7	−30.2 ± 5.7 ^#&^
LV-gLS (%)	−15.3 ± 2.1	−16.3 ± 2.4 *	−16.2 ± 3.1	−15.7 ± 2.1	−15.9 ± 2.3	−17.1 ± 3.2 ^‡^	−12.4 ± 1.5	−16.1 ± 1.4 ^#^	−19.9 ± 1.1 ^#&^

Abbreviations: LV-gRS = LV global radial strain, LV-gCS = LV global circumferential strain, LV-gLS = LV global longitudinal strain, LV-gAS = LV global area strain, LV-g3DS = LV global three-dimensional strain, LV = left ventricular. * *p* < 0.05 vs. LV-gRS < 16.1%, ^†^ *p* < 0.05 vs. 16.1% ≤ LV-gRS ≤ 34.7%, ^§^ *p* < 0.05 vs. LV-gCS < −22.6%, ^‡^ *p* < 0.05 vs. −22.6% ≤ LV-gCS ≤ −32.6%, ^#^ *p* < 0.05 vs. LV-gLS < −13.6%, ^&^ *p* < 0.05 vs. −13.6% ≤ LV-gLS ≤ −18.6%.

## Data Availability

The data are not publicly available due to ethical reasons.
